# A comparative study of the COVID-19 vaccine efficacy among cancer patients: mRNA versus non-mRNA

**DOI:** 10.1371/journal.pone.0281907

**Published:** 2023-03-01

**Authors:** Andhika Rachman, Anggraini Iriani, Lugyanti Sukrisman, Wulyo Rajabto, Nadia Ayu Mulansari, Anna Mira Lubis, Rahmat Cahyanur, Findy Prasetyawati, Dimas Priantono, Bayu Bijaksana Rumondor, Rachelle Betsy, Samuel Juanputra

**Affiliations:** 1 Division of Hematology and Medical Oncology, Department of Internal Medicine, Dr. Cipto Mangunkusumo General Hospital—Faculty of Medicine Universitas Indonesia, Jakarta, Indonesia; 2 Department of Clinical Pathology, Yarsi University, Jakarta, Indonesia; 3 Department of Internal Medicine, Dr. Cipto Mangunkusumo General Hospital—Faculty of Medicine Universitas Indonesia, Jakarta, Indonesia; University of Hail, SAUDI ARABIA

## Abstract

**Background:**

Cancer patients have an increased risk of a severe COVID-19 infection with higher mortality rate. This study aimed to analyze the levels of anti-SARS-CoV-2 S-RBD IgG and NAB among cancer patients who were vaccinated with COVID-19 vaccines, either with BNT162b2, mRNA-1273, AZD1222/ChAdOx1nCoV-19, or Coronavac/BBIBP-CorV vaccines.

**Method:**

A cross-sectional study was conducted among subjects with either solid or hematological cancers who had received two doses of either mRNA or non-mRNA vaccines within 6 months. The levels of anti-SARS-CoV-2 S-RBD IgG and NAb were analyzed using the Mindray Immunoassay Analyzer CL-900i. Statistical analysis was conducted using mean comparison and regression analysis.

**Result:**

The mRNA-1273 vaccine had the highest median levels of S-RBD IgG and NAb, followed by BNT162b, ChAdOx1nCoV-19, and BBIBP-CorV/Coronavac. The levels of S-RBD IgG and NAb in subjects vaccinated with mRNA vaccines were significantly higher than those of non-mRNA vaccines when grouped based on their characteristics, including age, type of cancer, chemotherapy regimen, and comorbidity (*p*<0.05). Furthermore, the S-RBD IgG and NAb levels between the subjects vaccinated with non-mRNA vaccines and the subjects vaccinated with mRNA vaccines were significantly different (*p*<0.05). However, there was no significant difference between the same types of vaccines. This study demonstrated a very strong correlation between the level of S-RBD IgG and the level of NAb (R = 0.962; *p*<0.001). The level of anti-SARS-CoV-2 S-RBD IgG was consistently higher compared to the level of NAb.

**Conclusions:**

Generally, mRNA vaccines produced significantly higher anti-SARS-CoV-2 S-RBD IgG and NAb levels than non-mRNA vaccines in cancer subjects.

## Introduction

Coronavirus Disease 2019 (COVID-19) is caused by severe acute respiratory syndrome coronavirus-2 (SARS-CoV-2), a virus that belongs to the *Betacoronavirus* genus. COVID-19 is known to have a wide spectrum of clinical manifestations, ranging from asymptomatic infection to respiratory failure. Cancer patients are at risk of a more severe COVID-19 infection with higher mortality rate. The Cancer Consortium Registry (CCC19) stated that the mortality rate of cancer patients with COVID-19 is 26% higher than the mortality rate of non-cancer patients with COVID-19. Cancer type, age, performance status, and comorbidities are some of the factors that may impact the clinical outcome of cancer patients infected with COVID-19 [1–3].

SARS-CoV-2 has several proteins in its outer surface, one of which is the spike (S) protein. The S protein is a large class I fusion transmembrane homotrimer protein that plays a big role in viral infectivity [3]. The N-terminal domain (NTD), receptor binding domain (RBD), and the conserved domains of the S2 subunit are all neutralizing antibody (NAb) epitopes of the S protein [4]. Through the receptor-binding domain (RBD), which is in the S1 domain of the S protein, SARS-CoV-2 is able to bind onto angiotensin converting enzyme (ACE)-2 receptor. It then fuses with the host membranes via the S2 subunit of the S protein which allows the viral RNA to enter the host cell’s cytoplasm [3]. The S protein also serves as a target for the host’s immunological response to induce the production of specific antibodies towards SARS-CoV-2.

The world’s vaccine-makers began to focus on the S epitope. Fortunately, the most potently neutralizing epitope, the RBD of the S epitope, is tremendously conserved and vaccines targeting this epitope may be capable to protect against all circulating SARS-CoV-2 strains. Additionally, vaccinations consistently induced high levels of NAb and IgG in all of the participants in recent studies [4]. Therefore, the NAb and S-RBD IgG have been widely used in phase I-III clinical trials COVID-19 vaccines to examine the efficacy, immunogenicity, as well as the optimal vaccine dose [4, 5].

In Indonesia, the most readily available vaccines are BNT162b2 and mRNA-1273, which are mRNA-based vaccines; AZD1222/ChAdOx1nCoV-19, which is a replication deficient adenoviral vector vaccine; and Coronavac/BBIBP-CorV, which is inactivated vaccine. These vaccines have been proven to be efficacious in the normal population based on some clinical trials [4]. However, the efficacy and immunogenicity of these vaccines for cancer patients need further investigation. Some studies have shown that cancer patients do not respond as well to these vaccinations, especially those receiving specific treatment regimens that impair the immune response [1, 2].

The essential world cancer organizations have recommended the COVID-19 vaccination for all patients with cancer, including those receiving active anticancer therapy. There is currently limited data available regarding the immunogenicity of these approved COVID-19 vaccines in cancer patients [6–10]. This research aims to evaluate the immune response of cancer patients towards several COVID-19 vaccines by measuring the levels of anti-SARS-CoV-2 S-RBD IgG and NAb. This research also aims to compare the S-RBD IgG and NAb levels between the cancer patients who received mRNA-based vaccines and the cancer patients who received non-mRNA-based vaccines.

## Material & method

### Research subjects

This was a multi-center cross-sectional study conducted at Dr. Cipto Mangunkusumo General Hospital and Pondok Kopi Islamic Hospital. The samples in this study were gathered for 6 months, from October 2021 to March 2022. The subjects were patients diagnosed with either solid or hematological cancers; aged ≥ 18 years old; had received two doses of COVID-19 vaccination; and within 6 months after completing two-doses of vaccination. Patients who already had their COVID-19 vaccine boosters were excluded.

### Sample collection and processing

Approximately 3 ml of venous blood sample was drawn and centrifuged at 4000 rpm for 10 minutes. The serum was then harvested and stored at -20°C for storage. The serum was examined by Chemiluminescent immunoassay or CLIA method to measure the levels of anti-SARS-CoV-2 S-RBD IgG antibody (S-RBD IgG) and anti-SARS-CoV-2 neutralizing antibody (NAb) using the Mindray immunoassay analyzer CL-900i. The results of both S-RBD IgG and NAb are measured in AU/mL. According to the assay manufacturer, the cut-off values for both SARS-CoV-2 NAb and S-RBD IgG seropositivity were >10 AU/mL.

### Statistical analysis

Subject characteristics are then presented in a table. The averages of numeric variables that are normally distributed were presented in the form of a mean and standard deviation (SD), whereas those that are not normally distributed were presented as a median and interquartile range (IQR). Significant differences between the means of two groups were analyzed using the independent-sample T test (for parametric data) or using the Mann-Whitney U test (for non-parametric data). One-Way ANOVA and post hoc test were done to compare significant differences between the means of antibody levels for each vaccine with a post hoc analysis using the Hochberg test. Afterward, a bivariate correlation analysis was measured between IgG and NAb using Kendall’s regression analysis.

### Ethical approval

Ethical approval for this study was granted by The Ethics Committee of The Faculty of Medicine, Universitas Indonesia (ethical approval number: KET–999/UN2.F1/ETIK/PPM.00.02/2021). This research was performed in accordance with the Declaration of Helsinki. Written informed consent was obtained from all subjects involved in the study.

## Results

A total of 119 patients were included in this study. All subjects had received two doses of either BNT162b2, mRNA-1273, AZD1222/ChAdOx1nCoV-19, or Coronavac/BBIBP-CorV vaccines without boosters. The majority of the participants were female (86.6%).

About 92.4% of the participants had solid organ cancer, which comprises gynecological, breast, lung, prostate, pancreatic, head and neck, brain, colorectal, kidney, and testicular malignancies. Only 7.6% of the subjects had hematologic malignancies, consisting of leukemia and lymphoma (Table 1).

**Table 1:** Subject characteristics.

Characteristics	N (119)
**Sex, N(%)**	
Male	16 (13.4)
Female	103 (86.6)
**Age, N(%)**	
≤ 60	99 (83.2)
> 60	20 (16.8)
**Cancer group, N(%)**	
Solid	110 (92.4)
Non-solid	9 (7.6)
**History of COVID-19 infection, N(%)**	
Yes	27 (22.7)
No	92 (77.3)
**Vaccine, N(%)**	
BNT162b2	32 (26.9)
mRNA-1273	15 (12.6)
AZD1222/ChAdOx1nCoV-19	19 (16)
Coronavac/BBIBP-CorV	53 (44.5)
**Vaccine type**	
mRNA vaccine	47 (39.5)
Non-mRNA vaccine	72 (60.5)
**Chemotherapy, N(%)**	96 (80.7)
Single agent	28 (29.1)
Combination	45 (46.9)
No data	23 (23.9)
**Time since last chemotherapy, N(%)**	
≤ 6 months	29 (24.4)
> 6 months	67 (56.3)
**Comorbidities**	
No	82 (68.9)
Yes	37 (31.1)
**Anti-SARS-CoV-2 antibody level (AU/mL)**	
S-RBD IgG, median [IQR]	270.56 [658.01]
NAb, median [IQR]	129.03 [225.61]
**Seroconversion S-RBD IgG, N(%)**	111 (93.3)
mRNA, N = 47	45 (95.7)
Non-MRNA, N = 72	66 (91.7)
**Seroconversion NAb, N(%)**	112 (94.1)
mRNA, N = 47	46 (97.9)
Non-mRNA, N = 72	66 (91.7)

Abbreviation: S-RBD IgG, spike protein’s receptor-binding domain immunoglobulin G; NAb, neutralizing antibody; IQR, interquartile range; SD, standard deviation

About 80.7% of the subjects received systemic chemotherapy, either exclusively or with concurrent radiotherapy. Chemotherapy regimens include hormonal therapies, anthracycline-based chemotherapy, taxane-based chemotherapy, alkylating agents, antimetabolite drugs, kinase inhibitors and topoisomerase inhibitors, monoclonal antibodies, vinca alkaloids, and steroids. These agents are either used exclusively or in combinations based on the National Comprehensive Cancer Network (NCCN) guidelines [11]. Subjects who had a history of myocardial infarction, congestive heart failure, peripheral vascular disease, stroke or transient ischaemic index, chronic obstructive pulmonary disease, connective tissue disease, peptic ulcer, liver disease, diabetes mellitus, and chronic kidney disease are considered to have comorbidities according to the Charlson Comorbidity Index (CCI) [12].

**Fig 1:**
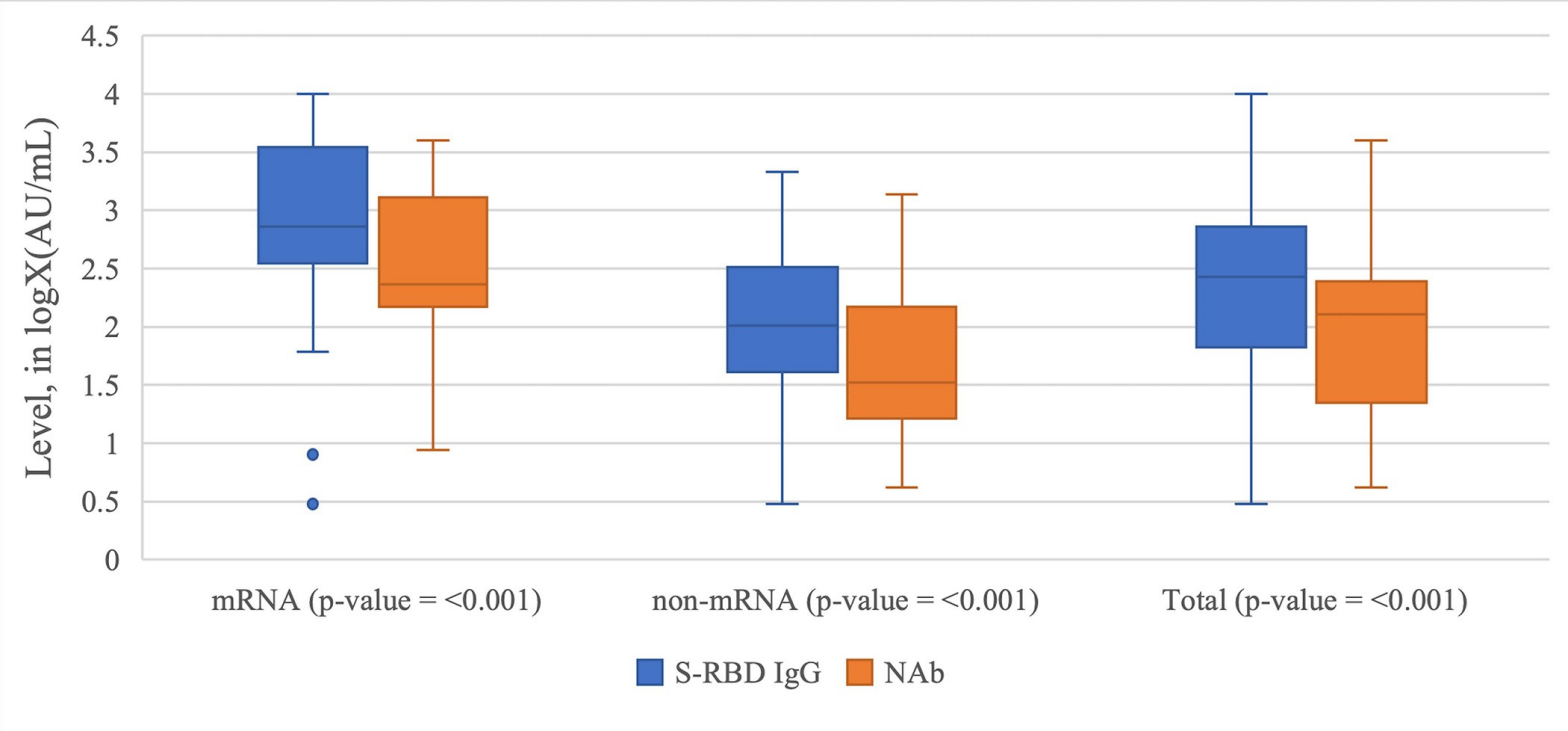
The Box Plot of NAb and S-RBD IgG level in logX(AU/mL) grouped by the vaccine types and cumulatively. The cut-off value for NAb and S-RBD IgG seroconversion is 10 AU/mL, which means that any data greater than or equal to log10 or 1 in the graph above achieves seropositive conversion.

As observed in Table 1 and Fig 1, the level of S-RBD IgG was consistently higher than the level of NAb. The levels of S-RBD IgG and NAb were also consistently higher among subjects vaccinated with mRNA vaccines than subjects vaccinated with non-mRNA vaccines. The seropositivity rate of both S-RBD IgG and NAb in subjects vaccinated with mRNA vaccines was higher than the seropositivity rate of S-RBD IgG in subjects vaccinated with non-mRNA vaccines.

In Table 2, there was a significant difference in S-RBD IgG levels between the four different vaccines. The NAb also showed a significant difference between the four different vaccines. The levels of S-RBD IgG and NAb are higher among the subjects vaccinated with mRNA vaccine when compared to the subjects vaccinated with non-mRNA vaccine (*p*< 0.001).

**Table 2:** Anti-SARS-CoV-2 S-RBD IgG antibody and anti-SARS-CoV-2 NAb levels.

Vaccine Type	S-RBD IgG (n = 119) (Median [IQR]) in AU/mL	NAb (n = 119) (Median [IQR]) in AU/mL
**Based on the vaccine type**		
mRNA	725.24 [3132.09]	232.70 [1143.19]
Non-mRNA	102.78 [284.53]	33.45 [132.18]
p value*	**<0.001** **	**<0.001** ^**+**^
**Based on the vaccine**		
BNT162b2	693 [3380.73]	224.68 [1717.37]
mRNA-1273	1441.10 [2010.76]	361.31 [893.67]
AZD1222/ChAdOx1nCoV-19	227.35 [451.68]	120.44 [175.99]
Coronavac / BBIBP-CorV	77.10 [223.13]	24.72 [115.23]
p value*	**<0.001** ^++^	**<0.001** ^+++^

*Paired t-test with transformation

**Independent t-test with transformation

^+^Mann-Whitney test

^++^ANOVA test with transformation

^+++^Kruskal-Wallis test

Abbreviation: S-RBD IgG, spike protein’s receptor-binding domain immunoglobulin G; NAb, neutralizing antibody; IQR, interquartile range; SD, standard deviation. The value in bold denotes statistical significance.

**Fig 2:**
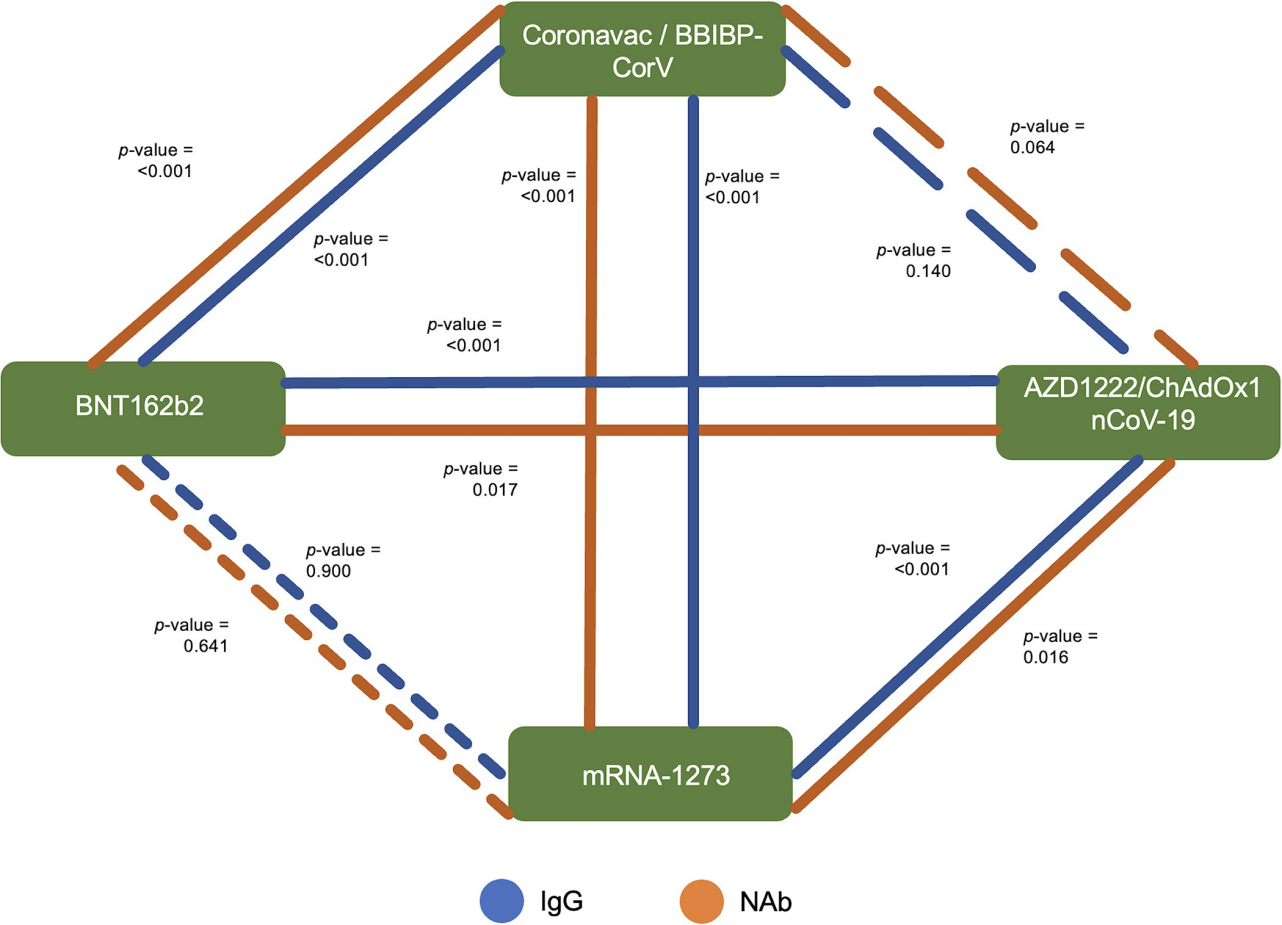
Horchberg’s GT2 post hoc multiple comparison tests of S-RBD IgG and NAb levels between vaccines. The blue line illustrates the S-RBD IgG significance between the vaccines, whereas the orange line illustrates the NAb significance. The continuous lines indicate a significant association, whereas the dashed lines illustrate the absence of a significant association. The sparser the line is, the less significant the association is.

In Fig 2, Horchberg GT2 post hoc analysis revealed that there’ significant difference in S-RBD IgG and NAb levels between non-mRNA-based vaccines (Coronavac/BBIBP-CorV and ChAdOx1nCoV-19) and mRNA-based vaccines (BNT162b2 and mRNA-1273) (*p*<0.05). However, there was no significant difference between the same types of vaccines (BNT162b2 vs. mRNA-1273; and Coronavac/BBIBP-CorV vs. ChAdOx1nCoV-19) (Fig 2).

**Fig 3:**
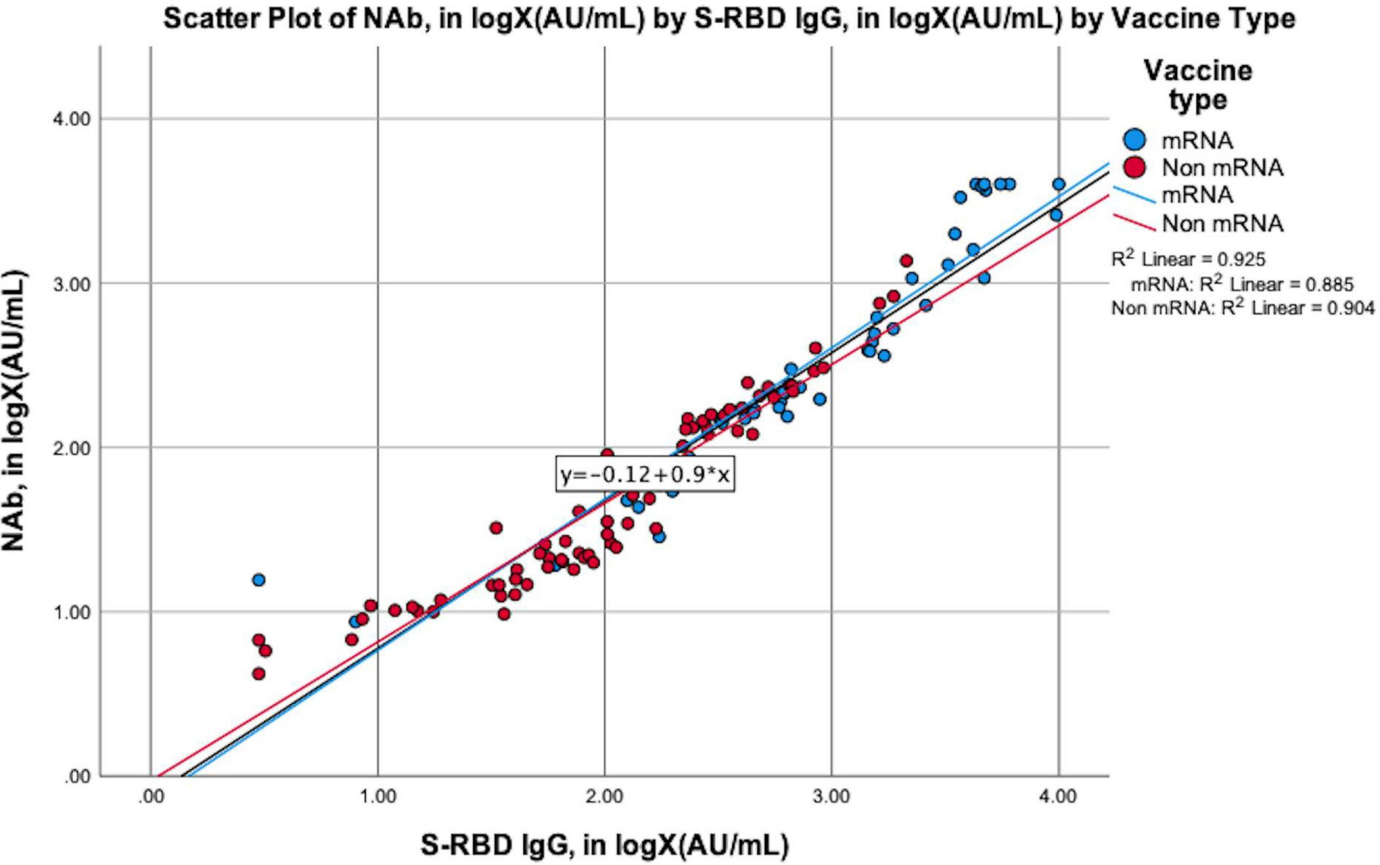
The scatter plot of NAb by S-RBD IgG, grouped by the types of vaccines and cumulatively. The blue dots and line in the graph illustrate the amount of NAb (in logX (AU/mL)) by S-RBD IgG (in logX (AU/mL)) for the subjects vaccinated with mRNA vaccines, whereas the red dots and lines illustrate the amount of NAb (in logX (AU/mL)) by S-RBD IgG (in logX (AU/mL)) for the subjects vaccinated with non-mRNA vaccines. The black line illustrates the amount of NAb (in logX (AU/mL)) by S-RBD igG (in logX (AU/mL)) for the cumulative group.

We also aimed to determine whether S-RBD IgG could be used as a proxy for NAb. As shown in Fig 3, the correlation between the levels of S-RBD IgG and NAb in both the total and the groupings based on the vaccine types (mRNA vaccines and non-mRNA vaccines) was very strong (R = 0.962; *p*<0.001). Both of the mRNA and non-mRNA vaccines had very strong correlations between the levels of S-RBD IgG and NAb (R = 0.941, *p*<0.001; R = 0.951, *p*<0.001, respectively).

From Table 3, we could see that in this study, subjects who received mRNA-based vaccines were able to produce higher S-RBD IgG and NAb levels compared to the subjects who received non-mRNA-based vaccines in almost every subgroup. However, there was no significant difference in the levels of S-RBD IgG and NAb in subjects over 60 years old and subjects whose last chemotherapy regimen was within 6 months of this study.

**Table 3:** Anti-SARS-CoV-2 antibody level comparison based on subject characteristics.

Characteristics	S-RBD IgG			NAb		
	mRNA	Non-mRNA	*p*-value	mRNA	Non-mRNA	*p*-value
	mean±SD or median[IQR] in AU/mL	mean±SD or median[IQR] in AU/mL		mean ±SD or median[IQR] in AU/mL	mean ±SD or median[IQR] in AU/mL	
**Age**						
< 60, N = 99	752.24 [3152.92] (N = 41)	104.46 [265.01] (N = 58)	**<0.001** ^**a**^	299.65 [1288.85] (N = 41)	33.45 [128.11] (N = 58)	**<0.001** ^**a**^
> = 60, N = 20	1240.75 ± 1656.41 (N = 6)	102.45 [352.24] (N = 14)	0.252^b^	141.82 [1359.33] (N = 6)	46.24 [166.10] (N = 20)	0.124^b^
*p*-value	0.293^a^	0.723^b^		0.264^b^	0.754^a^	
**Cancer Type**						
Hematologic, N = 9	2245.63 ± 2145.41 (N = 6)	5.18 ± 3.59 (N = 3)	**<0.001**	1255.56 ± 1535.98 (N = 6)	6.96 ± 3.5 (N = 3)	**<0.001**
Solid, N = 110	752.24 [2540.21] (N = 41)	106.11 [296.66] {N = 69)	**<0.001** ^**b**^	226.27 [1028.91] (N = 41)	35.42 [134.94] (N = 69)	**<0.001** ^**a**^
*p*-value	0.864^a^	**<0.001** ^**b**^		0.736^b^	**<0.001** ^**a**^	
**Prior COVID-19 infection**						
Yes, N = 27	3680.72 ± 2750.07 (N = 11)	283.96 [638.12] (N = 16)	**<0.001** ^**b**^	2200.85 ± 1598.99 (N = 11)	153.37 [229.09] (N = 16)	**<0.001** ^**a**^
No, N = 92	617.24 [8.08] (N = 36)	83.21 [229.71] (N = 56)	**<0.001** ^**c**^	200.01 [336.31] (N = 36)	25.20 [109.96] (N = 56)	**<0.001** ^**a**^
*p*-value	**0.005** ^**c**^	**0.002** ^**b**^		**0.002** ^**a**^	**<0.001** ^**a**^	
**Chemotherapy**						
Yes, N = 96	637.20 [2267.51] (N = 39)	102.13 [307.84] (N = 57)	**<0.001** ^**c**^	216.65 [921.92] (N = 39)	26.88 [139.43] (N = 57)	**<0.001** ^**a**^
No, N = 23	3213.11 ± 3175.92 (N = 8)	157.11 [294.50] (N = 15)	**<0.001** ^**b**^	1064.29 [3293.71] (N = 8)	70.32 [111.69] (N = 15)	**<0.001** ^**b**^
*p*-value	0.108^c^	0.597^b^		0.135^b^	0.537^a^	
**Chemotherapy regimen**						
Single agent, N = 28	662.13 [3050.36] (N = 13)	77.23 [92.50] (N = 15)	**<0.001** ^**b**^	229.65 [732.98] (N = 13)	26.88 [38.58] (N = 13)	**<0.001** ^**b**^
Combination, N = 45	456.89 [1381/18] (N = 19)	148.74 [380.05] (N = 26)	**0.002** ^**b**^	169.96 [559.35] (N = 19)	67.07 [147.68] (N = 26)	**0.002** ^**b**^
No data, N = 46						
*p*-value	0.173^b^	0.267^b^		0.299^b^	0.214^b^	
**Time** **since last chemotherapy**						
≤ 6 months, N = 29	556.64 [1413.67]	166.71 [465.84]	0.129^b^**	190.72 [322.86]	64.48 [204.71]	0.055^b^
> 6 months, N = 67	1490.72 [4266.80]	89.23 [242.30]	**<0.001** ^**b**^	412.57 [1302.20]	26.26 [124.44]	**<0.001** ^**a**^
*p*-value	0.109^b^*	0.988^b^		0.121^b^	0.97^d^	
**History of comorbidities**						
Yes, N = 37	1579.98 [3858.00] (11)	96.00 [237.25] (26)	**<0.001** ^**b**^	527.29 [3152.86] (11)	32.27 [118.11] (26)	**<0.001** ^**b**^
No, N = 82	649.67 [2763.25] (36)	109.28 [369.34] (46)	**<0.001** ^**b**^ **	215.40 [930.93] (36)	34.96 [153.57] (46)	**<0.001** ^**b**^
*p*-value	0.815^b^*	0.708^b^		0.307^b^	0.65^b^	

* 1 dataset was ignored due to being an outlier that challenge the normality of the data

** 2 dataset was ignored due to being an outlier that challenge the normality of the data

^a^Tested using Mann-Whitney U test

^b^Tested using t-test after data transformation into logX

^c^Tested using t-test after data transformation into (logX)^2^

^d^Tested using t-test after data transformation into -1/√X

Abbreviation: S-RBD IgG, spike protein’s receptor-binding domain immunoglobulin G; NAb, neutralizing antibody; IQR, interquartile range; SD, standard deviation.

However, cancer patients who had a history of COVID-19 infection developed a significantly higher antibody level for both mRNA and non-mRNA vaccines than non-infected subjects (Table 3). As a result, we excluded subjects who had a prior COVID-19 infection (Table 4). As can be seen in Table 4, mRNA-based vaccines produce higher S-RBD IgG and NAb levels compared to non-mRNA-based vaccines in almost every subgroup. The level of anti-SARS-CoV-2 NAb showed a significant difference between the mRNA and non-mRNA vaccines in subjects older than 60 years old. The level of anti-SARS-CoV-2 S-RBD IgG and NAb antibodies showed no such significant difference in subjects with hematologic cancer. Both anti-SARS-CoV-2 S-RBD IgG and NAb demonstrated a significant difference in subjects whose time since their last chemotherapy was less than 6 months.

**Table 4:** Anti-SARS-CoV-2 antibody level comparison based on subject characteristics after exclusion of subjects with prior COVID-19 infection.

	S-RBD IgG			NAB		
	mRNA	Non-mRNA	*p*-value	mRNA	Non-mRNA	*p*-value
	Mean ±SD or median[IQR] in AU/mL	Mean ±SD or median[IQR] in AU/mL		Mean ±SD or median[IQR] in AU/mL	Mean ± SD or median[IQR] in AU/mL	
**Cummulative, N = 92**	617.24 [1338.08] (N = 36)	83.31 [229.71] (N = 56)	**<0.001c**	200.01 [336.21] (N = 36)	25.19 [109.96] (N = 56)	**<0.001a**
**Age**						
< 60, N = 77	617.24 [1235.02] (N = 30)	85.12 [230.21] (N = 47)	**<0.001b**	208.70 [303.82] (N = 30)	24.72 [114.38] (N = 47)	**<0.001a**
≥60, N = 15	560.49 [2370.72] (N = 6)	134.53 ± 172.70 (N = 9)	0.134b	141.82 [1359.33] (N = 6)	26.88 [81.96] (N = 9)	**0.045b**
*p*-value	0.268b	0.346b		0.612b	0.514e	
**Cancer Type**						
Hematologic N = 7	2331.72 ± 2512.72 (N = 4)	5.18 ± 3.59 (N = 3)	0.161	1309.72 ± 1885.10 (N = 4)	6.96 ± 3.50 (N = 3)	0.289
Solid N = 85	617.24 [1198.76] (N = 32)	89.23 [235.06] (N = 53)	**<0.001b**	200.01 [284.53] (N = 32)	26.26 [112.20] (N = 53)	**<0.001a**
*p*-value	0.555b	**<0.001b**		0.649b	**0.002a**	
**Chemotherapy**						
Yes, N = 76	1375.30 [3621.83] (N = 30)	141.77 [233.35] (N = 46)	**<0.001b**	380.00 [2031.92] (N = 30)	52.41 [110.09] (N = 46)	**<0.001b**
No, N = 16	1406.69 ± 2134.89 (N = 6)	201.97 ± 351.91 (N = 10)	**0.005**	183.10 [284.07] (N = 6)	94.07 ± 210.43 (N = 10)	**<0.001a**
*p*-value	0.212c	0.469b		0.174b	0.264e	
**Chemotherapy regimen**						
Single agent, N = 22	646.45 [2010.76] (N = 11)	67.23 [94.85] (N = 11)	**<0.001b**	226.72 [566.40] (N = 11)	24.72 [25.41] (N = 11)	**<0.001b**
Combination, N = 35	433.68 [422.21] (N = 14)	102.13 [252.31] (N = 21)	**0.003c**	155.84 [163.17] (N = 14)	26.26 [107.24] (N = 21)	**0.005b**
Received no chemotherapy, N = 16						
*p*-value	0.065c	0.489b		0.102b	0.459b	
**Time since last chemotherapy**						
≤ 6 months, N = 22	456.89 [501.01] (N = 13)	67.23 [246.61] (N = 9)	**0.037c**	168.34 ± 120.25 (N = 13)	24.72 [120.95] (N = 9)	**0.036a**
> 6 months, N = 54	2066.72 ± 2644.02 (N = 17)	215.93 ± 383.43 (N = 37)	**0.011**	216.65 [759.81] (N = 17)	22.20 [106.78] (N = 37)	**<0.001e**
Received no chemotherapy, N = 16						
*p*-value	**0.031c**	0.442b		0.137a	0.567e	
**History of comorbidities**						
Yes, N = 31	725.24 [4515.25] (N = 9)	96.02 [238.50] (N = 22)	**0.001b**	232.70 [3129.49] (N = 9)	30.90 [118.11] (N = 22)	**<0.001b**
No, N = 61	596.94 [1274.69](N = 27)	66.33 [228.83] (N = 34)	**<0.001c**	190.72 [304.47](N = 27)	23.69 [107.91] (N = 34)	**<0.001e**
*p*-value	0.282c	0.627b		0.187b	0.588e	
**Days from Second Vaccination to Screening**						
≤90 days	641.83 [1648.90] (N = 30)	240.88 ± 228.85 (N = 12)	**0.002c**	209.95 [425.38] (N = 30)	82.23 [162.56] (N = 12)	**0.003b**
>90 days	365.02 ± 2200.78 (N = 6)	70.20 [170.56] (N = 44)	**0.022b**	119.05 ± 90.04 (N = 6)	21.81 [81.82] (N = 44)	**0.049e**
*p*-value	0.129c	0.240b		0.090b	0.128e	

* 1 dataset was ignored due to being an outlier that challenge the normality of the data

** 2 dataset was ignored due to being an outlier that challenge the normality of the data

^a^Tested using Mann-Whitney U test

^b^ Tested using t-test after data transformation into logX

^c^Tested using t-test after data transformation into (logX)^2^

^d^Tested using t-test after data transformation into -1/√X

^e^dTested using t-test after data transformation into log(logx)

**Fig 4:**
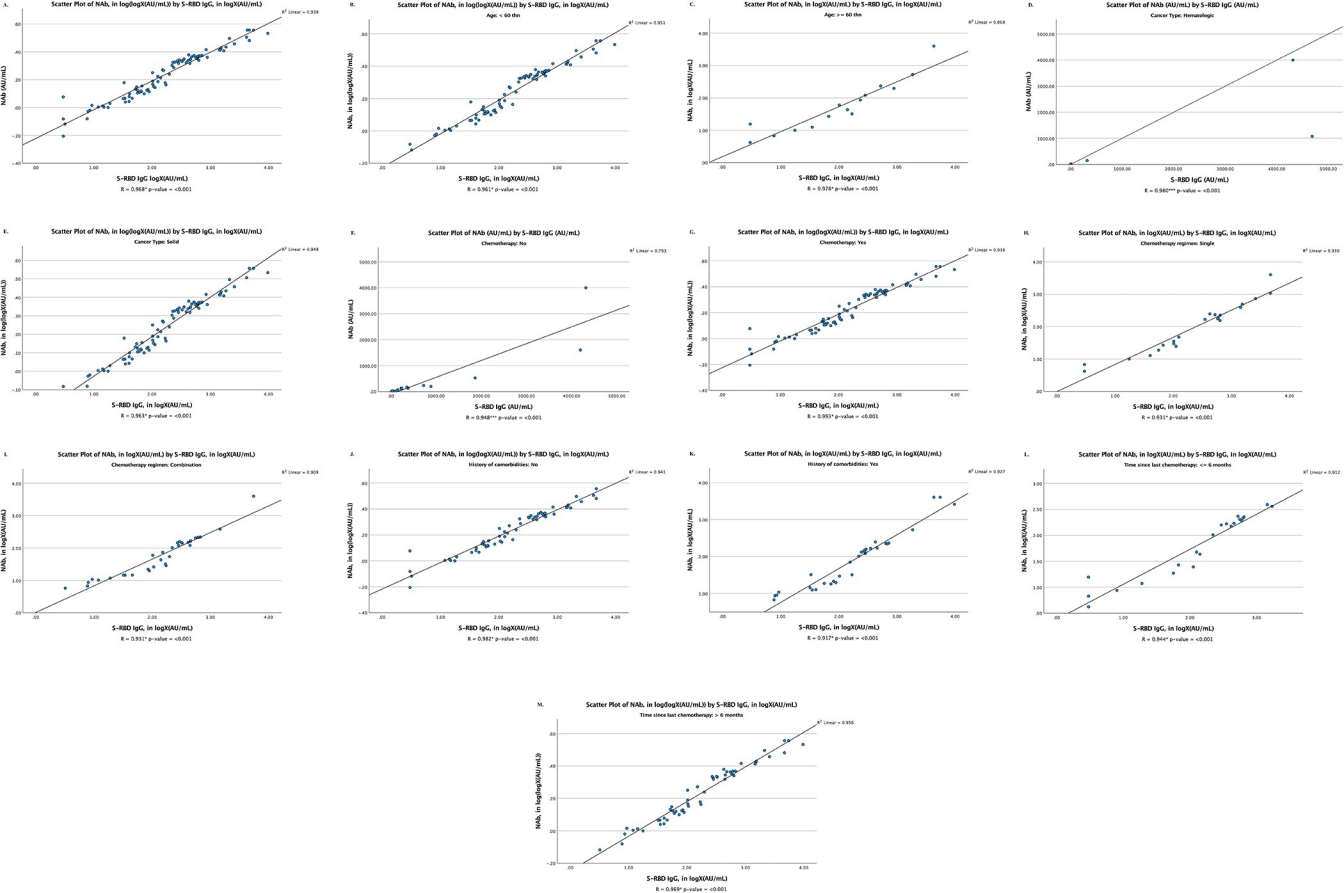
The scatter plots of S-RBD IgG in logX (AU/mL) by NAb in logX (AU/mL) in each subject’s characteristics who received BBIBP-CorV/Coronavac vaccines. (A) cumulative; (B) subjects less than 60 years old; (C) subjects over 60 years old; (D) subjects with hematologic cancer; (E) subjects with solid cancer; (F) subjects treated without chemotherapy; (G) subjects treated with chemotherapy; (H) subjects received single agent chemotherapy; (I) subjects received combination chemotherapy regimens; (J) subjects without a history of comorbidity; (K) subjects with a history of comorbidity; (L) time since last chemotherapy ≤6 months; (M) time since last chemotherapy >6 months. *Analyzed by Pearson correlation test after transformation into logX (AU/mL). **Analyzed by Kendall correlation test. ***Analyzed by Pearson correlation test. Abbreviation: S-RBD IgG, antibodies against receptor-binding domain of SARS-CoV-2 spike protein; NAb, neutralizing antibody.

As can be seen in Fig 4, the level of anti-SARS-CoV-2 S-RBD IgG demonstrated a very strong correlation with NAb in almost each cancer subject’s characteristics received inactivated vaccines.

## Discussion

In this research, we found that the median level of S-RBD IgG was consistently higher in all vaccines compared to NAb (Fig 1 and Table 1). This might be caused by the nature of the production of these antibodies. SARS-CoV-2 infection might also induce long-lived memory T-cells, memory B-cells, and mucosal-homing IgA plasmablasts [4, 13–15]. This is achieved through a long immune cascade elicited by an epitope held by the virus itself. Several known epitopes are the receptor binding domain (RBD), the N-terminal domain (NTD), and parts of the S2 subunit, all of which are parts of the S protein [4, 16, 17]. It was found that the strongest neutralizing epitope was the S-RBD, which was tremendously conserved in all strains of SARS-CoV-2, making it an ideal target for vaccine development [4].

The subjects who received mRNA-1273 and BNT162b2 vaccines in our study also had lower IgG levels compared to the subjects reported by Thakkar et al. (1441.10 [2010.76] vs. 11,963 [18,742] AU/mL and 224.68 [1717.37] AU/mL vs. 5,173 [16,699] AU/mL. However, Thakkar et al. performed the evaluation approximately 30 days after the complete vaccination dose. Their study also found statistically significant association between the time from vaccination until IgG testing and antibody levels [1]. Therefore, the NCCN recommends that cancer patients should receive the third dose of their primary vaccination series and an additional 2 booster doses because the anti-SARS-CoV-2 S-RBD IgG and NAb decrease gradually over time even after the second dose of COVID-19 vaccination [9].

In this study, when compared between the vaccine types, the subjects who received mRNA-based vaccine had significantly higher levels of S-RBD IgG and NAb compared to the subjects who received non-mRNA-based vaccine (Table 2). The subjects vaccinated with mRNA-1273 had the highest levels of S-RBD IgG and NAb, followed by subjects vaccinated with BNT162b2, ChAdOx1nCoV-19, and BBIBP-CorV/Coronavac.

Subjects who received Coronavac/BBIBP-CorV develop a lower titer of anti-SARS-CoV-2-RBD IgG and Nab compared to mRNA vaccines and AZD1222/ChAdOx1nCoV-19 vaccine. Inactivated-based vaccines work traditionally by activating APCs, which then induce CD4 T-cells in the presence of IFN- γ, resulting in the secretion of antibodies by B lymphocytes and the activation of CD8 T-cells, which lead to the apoptotic of infected cells.[18, 19] T-cell responses are essential to eliminate infected cells. CD4+ cells are crucial for B cell differentiation and the production of high-affinity antibodies in the germinal centers of secondary lymphoid organs [20]. The main outcome of the inactivated vaccine immunogenicity was the humoral immune response [21]. The T-cell responses induced by inactivated vaccines are poor and have been much less well studied than antibody-mediated immunity [22]. In contrast with mRNA and viral vector vaccines, which produce robust T cell and humoral immune responses following vaccination [20].

Several tremendous strategies have been designed for the mRNA vaccine. First, the 5’-capping is crucial to prevent exonuclease activity from degrading the mRNA, provide efficient pre-mRNA splicing, and provide a binding site for eIF4F, a component of the heterodimeric translation initiation complex [23–27]. Second, modifying the nucleosides in mRNA molecules can prevent the Toll-like receptors (TLRs) from recognizing RNA. Therefore, the RNA stability is improved by the nucleoside alterations [23, 28, 29]. Third, codon optimization improves translation efficiency, protein folding, and mRNA abundance. Codon optimization is critical for mRNA stability because the rate of codon-dependent translation elongation has been identified as a primary factor of mRNA stability [23, 30]. Fourth, the lipofectin-based carriers enhance the mRNA transport into target cells as well as protect the mRNA from RNase [23, 31, 32].

Furthermore, the mRNA vaccine also has a strategy that will induce more robust cellular expression of S via major histocompatibility complex (MHC) class I. The BNT162b1 vaccine optimizes their immunogenicity by encoding the trimerized RBD and producing stronger cellular immunity such as CD4+ and CD8+ T-cells. The mRNA-1273 vaccines, aside from inducing a robust CD8+ T-cell response, they also maintain the balance between the T helper type I (Th1) and T helper type II (Th2) responses [4, 33–35].

Those beneficial mechanisms made mRNA vaccines have a special strength, as it has been reported that they can stimulate not only humoral adaptive responses, but also cellular adaptive responses, including the activation of T helper cells and cytolytic T lymphocytes. Therefore, the diversity in vaccine design as well as the laboratory-proven potential to generate higher levels of S-RBD IgG and NAb make the mRNA vaccines hailed as the most promising vaccine candidates in the battle against the COVID-19 pandemic [23].

In viral vector-based vaccines, the vaccine particles will be identified by the innate immune response. Then, the APCs will interact with the MHC I and II molecules. These interactions activate the CD4 T-cells and the CD8 T-cells to produce IL2, IL12, TGF, and IFN-γ cytokines. Furthermore, the CD8 T-cells become memory T-cells. The Th2 then interact with the MHC II receptors on the surface of the B-cells, leading them to produce IL4, IL5, IL6, IL10, and TGF-β to stimulate B-cells activation and differentiation into plasma cells and memory B-cells. The plasma cell produces NAbs that are responsible for clearing the infection [36–39].

As can be observed in Fig 2, both the levels of S-RBD IgG and NAb show a significant difference between mRNA and non-mRNA-based vaccines. Several studies reported that the NAb contracted faster and earlier than the IgG. However, IgG levels reach a higher peak and have a longer lifespan than NAb [40–43]. A cohort study that was conducted by Terpos et al. showed that up to 6 months post-symptom onset (PSO), the estimated half-life for the S-RBD IgG and NAb were 62 and 47 days, respectively. After 6 months of infection, the estimated half-life for the S-RBD IgG and NAb were declining to 212 and 27 days, respectively [42]. A systematic review by Post et al. revealed that the IgG levels peaked 3–7 weeks post symptom onset, then plateaued and remained stable for at least 8 weeks [43].

The binding of NAb to the ACE-2 binding site on the RBD inhibits viral entry [3]. A study by Barnes et al. divided the NAb structure into several categories. First, the NAb that is generated by the VH3-53 gene section with short CDRH3 loops that bind exclusively to “up” RBDs and inhibit ACE-2; second, the NAb that inhibits the ACE-2 receptor and binds both “up” and “down” RBDs, as well as the adjacent RBD [44–46]; third, the NAb that identifies “up” and “down” RBD and binds outside the ACE-2 site; fourth, the antibody previously described that binds selectively to “up” RBD and does not inhibit the ACE-2 receptor [44, 47]. Category 2 consisted of four NAbs with RBD-bridging epitopes, including a VH3-53 antibody that spanned between two down RBDs using an extensive CDRH3 with a hydrophobic tip that preserved the spike in a closed configuration. The structures indicate an abundance of unexpected interactions between the NAb and the spike protein, most notably the antibody that has been identified to reach over adjacent RBD on the protomer of a single trimer. Additionally, the crystal structures of Fab-monomeric RBD complexes are beneficial for identifying the flexible "up" or "down" RBD configurations on the spike trimer that are targeted for neutralization [44].

Furthermore, not all antibodies are NAbs. Some are classified as binding antibodies. anti-SARS-CoV 2 was divided into NAb and non-neutralizing antibody (non-NAb) based on its effect. NAb is an antibody that inhibits the binding between pathogen and host (neutralization). NAb mainly refers to the receptor binding domain of S-protein (S-RBD), which prevents the binding of subunit to ACE-2 receptor [3, 48]. The term “neutralizing” refers to the antibodies’ ability to inhibit the initial pathogenic step by itself, making it crucial to achieve protection against SARS-CoV-2 [49]. Neutralization by NAbs can be achieved before the pathogen attachment to the host cells (by aggregation, immobilization, or destabilization), during the interference with the pathogen attachment (by physically blocking viral fusion to target cells), and during the post-attachment neutralization (inhibition of fusion and other steps) [3, 50–53]. Antibodies may also interact with some immune components and activate a complement cascade, which stimulates other immune responses such as cell lysis, lymphocyte recruitment, and antigen internalization. This leads to antibody-mediated pathogen clearance [3, 54]. Immunoglobulin (Ig) is a class of antibodies that comprises of IgA, IgM, and IgG, which has a role in neutralizing SARS-CoV-2. However, the maximum neutralization activity against SARS-CoV-2 is only accomplished when all three immunoglobulin classes (IgG, IgM, and IgA) are detected [3, 55].

As can be observed in Figs 3 and 4, the strong correlation between NAb and S-RBD IgG in inactivated, mRNA, and non-mRNA-based-vaccines has indicated that when the NAb cannot be detected, the S-RBD IgG can be detected instead. This is due to the synergetic characteristic of the S-RBD IgG that will also be in the high level as does NAb. However, when the IgG level is high, the NAb level may not also be high due to the maximum neutralization that has not been achieved by the increase in the IgG alone [3, 56–58].

A study by Ju et al. supports the findings of our data and concluded that the competition between NAb and ACE-2 may be a stronger indicator for the efficacy of virus-neutralizing antibodies than for the binding affinity. Therefore, inhibiting the interaction between RBD and ACE-2 receptor may serve as a suitable surrogate for neutralization. The anti-RBD antibodies are indicated to be predominantly viral species-specific inhibitors due to the crystal structure of RBD-bound antibody impeding the RBD’s binding to ACE-2, thus blocking the viral binding and entry [3, 58, 59]. This finding and explanation could be the answer to the unclear correlation between NAb and S-RBD IgG.

In this study, subjects less than 60 years old vaccinated with mRNA-based vaccines had the capability to produce higher S-RBD IgG and NAb levels compared to the subjects vaccinated with non-mRNA-based vaccines (Table 4). In subjects older than 60 years, the NAb was found to be significantly higher in mRNA group compared to non-mRNA group. However, there was no significant difference in the S-RBD IgG level in subjects over 60 years old. Evidence revealed that the S-RBD IgG level had higher sensitivity and specificity than NAb in evaluating the efficacy of COVID-19 vaccines [60]. These findings revealed that patients over the age of 60 had a poorer immune response to the vaccine than patients under the age of 60. Xia et al. found that the level of NAb in subjects over the age of 60 was lower than in people under the age of 60 [61].

Immunological system aging, or immunosenescence, is associated with a loss in immune function and prolonged activation of inflammation, which increase the vulnerability to viruses and decrease the responses to vaccination. Over time, aging also causes a gradual reduction of T-cells. The involution of the thymus is associated with alterations in the ratio of naïve T-cells to memory T-cells, which results in a greater proportion of memory T-cells in elderly people [62–65]. While T-cells tend to experience more extensive alterations as they age whereas B-cells only undergo gradual alterations as they age [66, 67]. The overall amount of B-cells decreases gradually with age, both in the periphery and bone marrow [62, 68].

In subjects with solid malignancies, the levels of S-RBD IgG and NAb antibodies were statistically significantly higher in the subjects receiving the mRNA vaccines compared to non-mRNA vaccines (Table 4). However, the levels of S-RBD IgG and NAb showed no significant differences among subjects with hematologic malignancies. Thakkar et al. revealed that subjects with hematologic malignancies had a significantly lower seropositivity rate compared to the subjects with solid tumors (85% vs. 98%, p = 0.001). They also reported that the IgG levels were significantly lower in the subjects with hematologic malignancies compared to the subjects with solid tumors (7,858 AU/mL, SD 18,103 vs. 2,528 AU/mL, SD 12,338, *p* = 0.013) [1]. Therefore, patients with hematologic cancers had higher immunosuppression conditions due to their immune system impairment, intrinsic frailty, or cancer therapies that can lead to significant lymphodepletion and myelosuppression. These conditions also led to a worse immune response to vaccination and increased the morbidity and mortality rate. As a result, individuals with haematological malignancies remain a high-risk population until the efficacious vaccines are invented [69–72].

In Table 4, three subjects with hematologic cancer showed no response to non-mRNA vaccines. They were the same subjects who received B-cell depleting therapy (rituximab). There is evidence that B cell-depleting therapy with rituximab (RTX) affects the humoral immune response after vaccination [73]. In a cohort study of subjects with hematologic and solid malignancies, those who received anti-CD20 therapy developed no antibody response. A longitudinal study of anti-spike revealed that B-cell targeted therapies were associated with reduced peak and sustained antibody responses [74]. In response to specific stimuli, B cells have the potential to regulate CD8+ T cell responses. Depletion of B cells decreases the efficacy of the cellular response, which includes cytotoxic T cells [75]. B cells play an important role in the development of humoral immunity. Mrak et al. found that the S-RBD antibody correlates with the number of B cells in the peripheral blood, implying that the presence of B cells is required for mounting a humoral response. SARS-CoV-2 RBD-specific antibodies showed a strong correlation with neutralizing activity, providing evidence for a protective antibody response. Their findings concluded that peripheral B cells are essential for both the quality and quantity of the humoral response following COVID-19 vaccination. Non-seroconverted patients have impaired humoral immunity due to the lack of peripheral B cells [73].

This research also demonstrated that the subjects with either single or combination chemotherapy vaccinated with mRNA vaccine also had greater levels of NAb and S-RBD IgG compared to the subjects with non-mRNA vaccines (Table 4). There is a concern that the cytotoxic chemotherapy will reduce the efficacy of SARS-CoV-2 vaccines in eliciting the humoral immune response due to these drugs affecting the bone marrow and suppresses the immune system. Interestingly, multiple studies revealed that cancer patients receiving chemotherapy had lower antibody titers compared to healthy subjects [76–79]. Cytotoxic chemotherapy can alter the DNA synthesis, replication, and cell cycle progression of rapidly proliferating lymphocytes upon immunological activation.[69, 80]. However, in this study, the COVID-19 vaccines were proven to be efficacious among the cancer patients treated with either single or combination chemotherapy (Table 4).

Subjects who received COVID-19 vaccination with the last time chemotherapy ≤ 6 months and > 6 months showed no such significant difference in S-RBD IgG and NAb levels. The optimal timing of vaccine administration for patients who received chemotherapy has not been established by the guideline yet. Some recommendations in certain circumstances were established. Patients already on cytotoxic chemotherapy recommended to get vaccines in between chemotherapy cycles. Patients completing cytotoxic therapy recommended to be vaccinated after therapy is completed. Further studies are required to fill these gaps and larger studies that include cancer patients are warranted to have a better understanding of the optimal timing of the vaccinees in cancer patients [81].

Aditionally, in this study, the levels of NAb and S-RBD IgG were not significantly different between patients with and without comorbidities. This finding was in line with a study by Dundar et al., which showed there was no significant correlation between comorbidities and antibody levels post COVID-19 vaccinations [82].

In groups ≤ 90 days and > 90 days from second vaccination to screening, m-RNA based vaccines produced S-RBD IgG and NAb at a significantly higher levels than non-mRNA based vaccines (Table 4). T-test analysis demonstrated that there was no significant difference in S-RBD IgG and NAb levels between < 90 days and ≥ 90 days. A cohort study by Campo et al. demonstrated that antibodies remained persistent 6 months after receiving two doses of the COVID-19 vaccines [83]. A study by Doria-Rose et al., also revealed that antibodies were still persistent 6 months after receiving the second dose of the mRNA-1273 vaccination [84]. Another study by Lai et al. showed the antibody had no significant difference when comparing two time points at months 2 and 6 after second doses of COVID-19 vaccines [85]. These studies were in line with our study, which collected the samples within 6 months due to antibody could persist for 6 months after receiving the second dose of COVID-19 vaccines.

This cross-sectional study has several limitations. This study has potential confounders which affect the outcome of the study. One of the influential confounders is the heterogenous types of cancer with each type having different cancer treatments and prognoses that may affect the efficacy of COVID-19 vaccines. However, despite this confounding factor, the mRNA vaccines proved to be highly effective among cancer patients. The sample acquisition time was considered as the limitation of the study due to the fact that during the recruitment, most cancer patients had received two doses of COVID-19 vaccines more than 3 months before the study was held. The COVID-19 national mass vaccination program in Indonesia commenced at February 2021. This study recruitment process started in October 2021. At that time, national COVID-19 vaccination coverage had reached 75%, which it became unreachable to recruit recently vaccinated cancer patients at specific time point. Furthermore, there was no healthy control group as reference population. This should be considered to improve the design and participants selection in further studies.

## Conclusion

Generally, mRNA-based vaccines produced significantly higher levels of S-RBD IgG and NAb than non-mRNA-based vaccines when compared across all subject characteristics. The levels of S-RBD IgG were consistently higher in all vaccines compared to the levels of NAb. There was a significantly positive strong correlation between S-RBD IgG and NAb levels among cancer patients who had received either mRNA or non-mRNA-based vaccines.
